# Peripherical Electrical Stimulation for Parkinsonian Tremor: A Systematic Review

**DOI:** 10.3389/fnagi.2022.795454

**Published:** 2022-02-07

**Authors:** Lin Meng, Mengyue Jin, Xiaodong Zhu, Dong Ming

**Affiliations:** ^1^Academy of Medical Engineering and Translational Medicine, Tianjin University, Tianjin, China; ^2^Department of Neurology, Tianjin Medical University General Hospital, Tianjin, China; ^3^Department of Biomedical Engineering, Tianjin University, Tianjin, China

**Keywords:** Parkinson's disease (PD), electrical stimulation, functional electrical stimulation (FES), neuromuscular electrical stimulation (NMES), peripheral nerve stimulation (PNS), tremor suppression

## Abstract

Parkinsonian tremor is one of the most common motor disorders in patients with Parkinson's disease (PD). Compared to oral medications and brain surgery, electrical stimulation approaches have emerged as effective and non-invasive methods for tremor reduction. The pathophysiology, detection and interventions of tremors have been introduced, however, a systematic review of peripherical electrical stimulation approaches, methodologies, experimental design and clinical outcomes for PD tremor suppression is still missing. Therefore, in this paper, we summarized recent studies on electrical stimulation for tremor suppression in PD patients and discussed stimulation protocols and effectiveness of different types of electrical stimulation approaches in detail. Twenty out of 528 papers published from 2010 to 2021 July were reviewed. The results show that electrical stimulation is an efficient intervention for tremor suppression. The methods fall into three main categories according to the mechanisms: namely functional electrical stimulation (FES), sensory electrical stimulation (SES) and transcutaneous electrical nerve stimulation (TENS). The outcomes of tremor suppression were varied due to various stimulation approaches, electrode locations and stimulation parameters. The FES method performed the best in tremor attenuation where the efficiency depends mainly by the control strategy and accuracy of tremor detection. However, the mechanism underlying tremor suppression with SES and TENS, is not well-known. Current electrical stimulation approaches may only work for a number of patients. The potential mechanism of tremor suppression still needs to be further explored.

## Introduction

Parkinson's disease (PD) is a progressive degenerative disease characterized by a substantial loss of dopaminergic neurons in in the substantia nigra resulting in motor dysfunctions (Tysnes and Storstein, 2017; Galvez et al., 2018), such as tremor, bradykinesia, posture instability and gait difficulties (Chapuis et al., 2005; Ly et al., 2016; Carvajal-Castano et al., 2021; Zhang et al., 2021). Although PD patients could exhibit various motor symptoms, studies showed that 69% of patients have resting tremor at the onset of the disease, and 75% develop tremor during the course of the disease (Hughes et al., 1993; Jankovic, 2008). Tremors usually occur in the hands and have a significant effect on daily life (Louis and Machado, 2015).

Levodopa is considered mostly effective for PD tremor in combination with carbidopa (Marjama-Lyons and Koller, 2000; Fishman, 2008). However, the efficiencies of medication are various among patients. It may even worsen in some patients (Fahn et al., 2004; Hallett, 2012). Invasive techniques, such as deep brain stimulation (DBS), has been proven effective for tremor reduction (O'Connor and Kini, 2011). But brain surgery has high risk and requires appropriate surgical indications (Okun et al., 2005; Hariz et al., 2008; Bronstein et al., 2011).

Conventional physical approach is considered as an alternative to oral medications and surgery where adding weights (McGruder et al., 2003), cooling joints (Cooper et al., 2000; Feys et al., 2005) and upper limb orthoses (Aisen et al., 1993; Fromme et al., 2020), have been used for functional tremor suppression. Studies have demonstrated the effectiveness of biomechanical loading approach for tremor reduction (Aisen et al., 1993; Fromme et al., 2020). However, there are still challenges for these orthoses utilized in clinical or home use due to limitations of wearability of the orthoses and human-computer interfaces (Nguyen and Luu, 2021).

The pathological tremor can be suppressed by regulating the neuronal pathway or controlling opposite muscles to preserve the voluntary movement as the deficit of the cerebellar feedforward control of voluntary movement may cause tremor (Fishman, 2008). The regulation of neuronal pathway can be achieved by peripheral nerve electrical stimulation (Raethjen et al., 2000; Xu et al., 2016; Dideriksen et al., 2017) while controlled muscle contraction can be generated by functional electrical stimulation (FES) (Javidan et al., 1990). Both methods have been proven their efficiencies in alleviating tremor (Javidan et al., 1992; Maneski et al., 2011). Peripherical electrical stimulation (PES) may be a more wearable and comfortable alternative for tremor suppression due to its unique advantages, such as non-invasive, light weight and low cost.

A few studies have reviewed the pathophysiology, detection and interventions of tremors, particularly PD tremor in details (O'Connor and Kini, 2011; Ly et al., 2016). However, to the authors' knowledge, there is no systematic review on electrical stimulation approaches, comparison of methodologies and clinical outcomes for Parkinsonian tremor suppression. In this paper, we will summarize the state of the art of peripherical electrical stimulation approaches for tremor reduction in recent 10 years. The article will provide a literature review of relevant studies from perspectives including tremor identification, electrical stimulation control strategies, experimental protocols and results from clinical treatments, and discuss potential research directions for improving the technique in future.

## Methods

A literature search was conducted using four databases: Web of Science, PubMed, Embase, and the Institute of Electrical and Electronics Engineers (IEEE) Xplore. Four groups of literature search terms were used, among which three groups are an “AND” relationship: (i) “Parkinson's disease” or “PD”; (ii) “neuromuscular electrical stimulation” or “NMES” or “electrical stimulation” or “functional electrical stimulation” or “FES” or “peripheral nerve stimulation” or “PNS”; (iii) “Tremor”; and a group of keywords was set for literature exclusion: (iv) “DBS” or “Deep brain stimulation.” Articles whose titles and/or abstracts meet the search strategy (i) AND (ii) AND (iii) NOT (iv) were included in this review. In addition to searching the electronic databases, a targeted search was also carried out on bibliographies of related articles to identify any other papers for inclusion.

Only original, full-text research articles published in English between January 2010 and July 2021 that investigate the suppression of tremor in PD patients using PES were considered in this review. Articles were excluded if they: (i) did not use peripherical electrical stimulation; (ii) only studied on other types of tremors (e.g., essential tremor (ET), hyperthyroidism, drug tremor) or patients with other diseases; (iii) were review articles.

## Results

### Search Results

**Figure 1:**
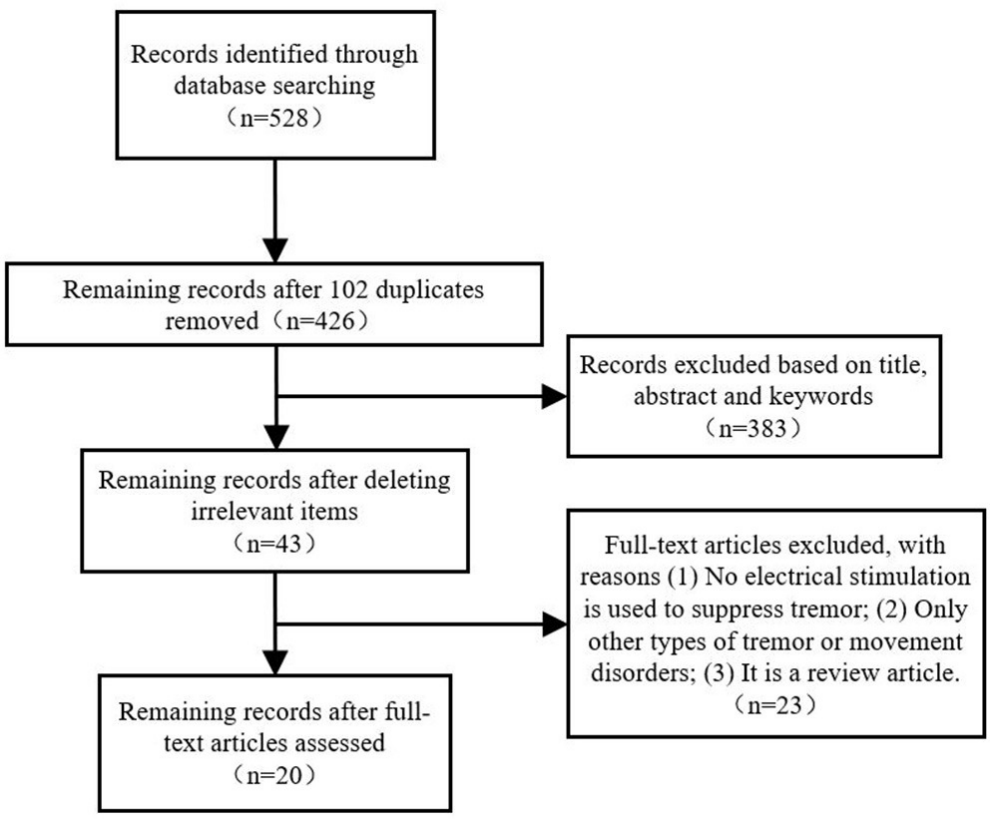
PRISMA flow chart of the screening and exclusion process.

Figure 1 shows a PRISMA flowchart that illustrates the screening and exclusion process. A total of 282 articles were retrieved from the Web of Science database while 158, 67, and 21 articles obtained from the PubMed, Embase and IEEE database, respectively, with the use of the above retrieval method. After duplicate and unrelated papers were removed after initial screening, 23 articles were excluded according to the criteria: 8 articles were excluded according to criterion (i), 10 articles were excluded according to criterion (ii), and 5 articles were excluded according to criterion (iii). A total of 20 articles were included in this review.

### Participants

Participants, strategies, stimulation locations, stimulation parameters, experimental protocols, and outcomes of the 20 studies have been summarized in this review as shown in Tables 1–3. Among all the reviewed articles, there were 9 papers that only recruited PD patients, and 7 papers that enrolled PD patients and healthy people or patients with other diseases for comparison. The remaining of 4 papers only included healthy subjects where tremor activities were physically imitated or artificially induced during the experiment.

**Table 1:** Summary of the methodologies and outcomes of functional electrical stimulation (FES).

**Reference**	**Participants**	**Strategies**	**Stimulation locations**	**Stimulation parameters**	**Experiment protocol**	**Outcomes**
Zhang and Ang (2007)	1 healthy participant	Out-of-phase	Biceps and triceps	A: 20 mA or 8–20 mA; PW: 100 μs; F: 20 Hz; single-phase pulse	Voluntary exercises and tremor exercises; EMG signal recorded	Surface EMG was used as feedback to adjust FES to suppress tremor without affecting voluntary movement.
Zhang et al. (2011)	6 PD; 3 other	Out-of-phase	Wrist joint: ECR and FCU; elbow joint: biceps and triceps	F:20 Hz; A:30 mA; PW: variable.	-	An average tremor suppression of about 90%
Maneski et al. (2011)	5 healthy participants; 4 PD; 3 ET	Out-of-phase	FCE and ECR; biceps and triceps	PW: 150 μs; A: 5–25 mA; F: 40 Hz; Bi-phase pulse	Rest tremor with arm resting on a chair support; 60s/trial with interval 3s stimulation on and 1s pause, 3 trials; angular velocity recorded	The average inhibition rate of all participants was 61 ± 7%
Alvaro Gallego et al. (2013a,b)	6 PD; 4 ET/3 PD; 9 ET	Co-contraction	FCR and ECU/FCR and ECU; biceps and triceps	F: 30 or 40 Hz PW: 250 or 300 μs	Finger to nose test; 30–35s/trial, 6–12 trials; angular velocity recorded	The tremor amplitude was decreased by 52.33 ± 25.48% on average
Freeman et al. (2015)	9 healthy participants	Co-contraction	ECR and FCR	F: 40Hz; A: variable; PW: 0–200 μs. Bi-phase pulse	Induced tremor; joint angle signal recorded	At all frequencies (2.5, 3, and 4 Hz), the amplitude of the single-peak frequency was reduced by more than 50%.
Castro et al. (2017)	3 PD	Out-of-phase	Wrist extensor or flexor muscle	-	Three sets of actions: arms extended, Grabbing a cup, Pinch; EMG signals recorded	FES can significantly suppress tremor
Copur et al. (2019)	4 healthy participants	Co-contraction	ECR and FCR	F: 40 Hz; PW: 300 μs; Bi-phase pulse	Induced tremor; 40s/trial, rest for 5 min; joint angle recorded	RC-FES had a better effect on suppressing tremor with the use of ZPHP filter to reduce the stimulation interference to voluntary movement

*PD, Parkinson's disease; ET, Essential tremor; FCR, Flexor carpi radialis; ECR, Extensor carpi radialis; ECU, Extensor carpi ulnaris; A, Amplitude; F, Frequency; PW, Pulse Width; RC, Repeat control*.

**Table 2:** Summary of the methodologies and outcomes of sensory electrical stimulation (SES).

**Reference**	**Participants**	**Strategies**	**Stimulation locations**	**Stimulation parameters**	**Experiment protocol**	**Outcomes**
Dosen et al. (2015)	4 PD; 2 ET	out-of-phase	Flexors and extensors of the wrist and fingers	F: 100 Hz; PW: 300 μs; A: variable	Resting posture with the arm rest on a chair support; 120s, stimulation was intermittently on and off; EMG signals recorded	The average tremor suppression levels were 60 ± 14% and 42 ± 5%, respectively, when the stimuli amplitude above and below the MT.
Dideriksen et al. (2017)	5 PD; 4 ET	out-of-phase	FCR and ECR	F: 100 Hz; PW: 400 μs;	Resting posture with the arm rest on a chair support; 150s/trial, the stimulation was on and off within a 30s window, 10 trials; EMG signals recorded	The method achieved the average tremor suppression level of 0.58 ± 0.35 across all patients.
Jitkritsadakul et al. (2015, 2017)	34 PD/30 PD	continuous	abductor pollicis brevis and interosseous muscle	F: 50 Hz; PW: 150 μs; A: < 20 Ma.	Trial time unknown, stimulation-ON for 10s; angular velocity recorded	The average percentage of improvement of the peak magnitude and RMS angular velocity was 49.57 and 43.81%, respectively. The tremor gloves effectively inhibit resting tremor in PD patients.
Heo et al. (2018, 2019)	14PD/14 PD; 9 SWEDDs	continuous	FCR, ECR and ECU	PW: 300 μs; F: 100 Hz; A: variable; Single-phase pulse	Resting posture with the hands supine on laps; 15s/trial, Pre Stim (5s)- Stim ON (5s)-Post Stim (5s), 3 trials; angular velocity recorded	The SES had no effect on the resting tremor of SWEDDs patients, but it significantly reduced the amplitude and peak frequency of PD patients. And the SES reduced the PD patients' tremor by an average of 53–68%.

*PD, Parkinson's disease; ET, Essential tremor; SWEDDs, Scans Without Evidence of Dopaminergic Deficits; MT, Motor threshold; FCR, Flexor carpi radialis; ECR, Extensor carpi radialis; ECU, Extensor carpi ulnaris; A, Amplitude; F, Frequency; PW, Pulse Width*.

**Table 3:** Summary of the methodologies and outcomes of transcutaneous electrical nerve stimulation (TENS).

**Reference**	**Participants**	**Strategies**	**Stimulation locations**	**Stimulation parameters**	**Experiment protocol**	**Outcomes**
Hao M.-Z. et al. (2013)	11 PD	Continuous	Radial nerve	F: 100 Hz; PW: 200 μs; A: 6 mA; Bi-phase pulse	Resting posture with the arm rest on a chair support; 40s, Pre Stim (10s) - Stim ON (20s) Post Stim (10s); joint angle and EMG signal recorded	The amplitude of shoulder tremor and the EMG of the hand muscles were significantly reduced while no EMG reduction was observed in the biceps and triceps
Xu et al. (2016)	3 healthy participants; 2 PD	Continuous	Radial nerve	A: 0–12 mA; F: 250 Hz; PW: 200 μs; Bi-phase pulse	The forearms on the table; 15s/trial, Pre Stim (5s)—Stim ON(5s)—Post Stim (5s), 10 trials; joint angle and EMG signal recorded	All four joint angles showed reduced tremor; Partial EMG suppression was reduced
Hao et al. (2017)	8 PD	Continuous	Radial nerve	F: 250 Hz; PW: 200 μs; A: variable Bi-phase pulse	The forearms on the table; 15s/trial, Pre Stim (5s)—Stim ON(5s)—Post Stim (5s), 9–13 trials; joint angle and EMG signal recorded	The average inhibition rate of joint tremor across all participants was 61.56%, and the average inhibition rate of myoelectricity was 47.97%.
Hu Z. et al. (2019) and Hu Z.-X. et al. (2019)	3 PD/10 PD	Continuous	Radial nerve	PW: 200 μs; F: 250 Hz. Bi-phase pulse	Do voluntary exercises after 2s stimulation with an audio stimulus; 5s/trial, 15–20 trials; EMG signals recorded	In addition to suppressing tremor, the TENS also reduced the speed of voluntary movement, but it did not prevent or interrupt voluntary movement.
Pascual-Valdunciel et al. (2019)	10 healthy participants	Continuous	Median nerve and Radial nerve	A: variable.	The forearms on the table; two stimulation intensity < MT and >MT: Each one consisting on sequences of 30 stimuli, 2 ± 0.2s ISI, 1 min rest, 3 trials	Inhibition of ECR: < MT/14 ± 5% of maximum M-wave; > MT/27 ± 9% of maximum M-wave; Inhibition of FCR: < MT/57 ± 13% of maximum M-wave; >MT/75 ± 12% of maximum M-wave.

*PD, Parkinson's disease; ET, Essential tremor; MT, Motor threshold; FCR, Flexor carpi radialis; ECR, Extensor carpi radialis; ECU, Extensor carpi ulnaris; A, Amplitude; F, Frequency; PW, Pulse Width; ISI, inter-stimulus interval*.

### Electrical Stimulation for Tremor Suppression

The electrical stimulation methods can fall into three main categories: namely FES, sensory electrical stimulation (SES) and transcutaneous electrical nerve stimulation (TENS). The three types of PES approaches have different mechanism for tremor suppression. The FES method induces muscle contraction to modulate its intrinsic property for suppressing tremor (Zhang and Ang, 2007; Maneski et al., 2011; Zhang et al., 2011; Alvaro Gallego et al., 2013a; Freeman et al., 2015; Castro et al., 2017; Copur et al., 2019). On the other hand, the SES also applies electrical current to the targeted muscle but with a stimulation amplitude below the motor threshold (Dosen et al., 2015; Dideriksen et al., 2017). The TENS method stimulates the afferent nerve (e.g., the radial and median nerves) to elicit cutaneous afferent fibers and inhibit tremor-related muscles (Hao M.-Z. et al., 2013; Xu et al., 2016; Hao et al., 2017; Hu Z. et al., 2019; Hu Z.-X. et al., 2019; Pascual-Valdunciel et al., 2019). The FES method was utilized in 8 studies where the flexor carpi radialis (FCR) and extensor carpi radialis (ECR) were electrically stimulated for suppressing wrist joint tremor (Maneski et al., 2011; Zhang et al., 2011; Freeman et al., 2015; Copur et al., 2019) and the biceps and tricpes brachioceps for the elbow joint (Zhang and Ang, 2007; Maneski et al., 2011; Zhang et al., 2011). Six studies employed the SES to the FCR and ECR with a higher stimulation frequency and current pulse width compared to the FES (Dosen et al., 2015; Dideriksen et al., 2017; Heo et al., 2018, 2019). A stimulation intensity below motor threshold was used for the SES to elicit the afferent pathway instead of activating muscle contraction (Jitkritsadakul et al., 2015, 2017). The remaining 6 studies stimulated the afferent nerve (e.g., radial and median nerves) in order to elicit afferent fibers and inhibit tremor-related muscles, so called TENS, and the highest stimulation frequency and pulse width were used compared to the FES and SES. Various control strategies have been developed for tremor suppression in which tremor detection is an essential element. Electromyography (EMG) signals and inertial data have been commonly used to identify tremors and assess the rate of tremor suppression (Zhang and Ang, 2007; Dosen et al., 2015; Castro et al., 2017; Dideriksen et al., 2017). Some studies utilized the combination of EMG and inertial data to distinguish between tremor and voluntary movement (Hao M.-Z. et al., 2013; Xu et al., 2016; Hao et al., 2017; Hu Z. et al., 2019; Hu Z.-X. et al., 2019). From the results, the tremor inhibition rate of FES exceeded 50% over all 8 studies, and the inhibition rates of the SES and TENS were close to ~50%. The stimulation strategies, stimulation locations and parameters, tremor detection algorithm and experiment protocols are further discussed while the clinical outcomes and potential mechanisms of the three PES types are analyzed in the following section.

## Discussion

This review summarized recent studies on the treatment of tremor suppression in PD patients by PES between January 2010 and July 2021. The methods are categorized into FES, SES and TENS. Electrical current is usually applied to pairs of antagonistic muscles (e.g., FCR and ECR) for the FES and SES and radial nerves for the TENS. Three stimulation strategies have been often employed, namely heterogeneous, co-contraction, and continuous electrical stimulation while stimulation parameters are various corresponding to different stimulation approaches. The inconsistency in stimulation model and experiment protocol can result in different clinical outcomes for tremor suppression.

### Electrical Stimulation Strategies

Regardless of PES types, three stimulation strategies are commonly used: out-of-phase, co-contraction and continuous stimulation.

**Figure 2:**
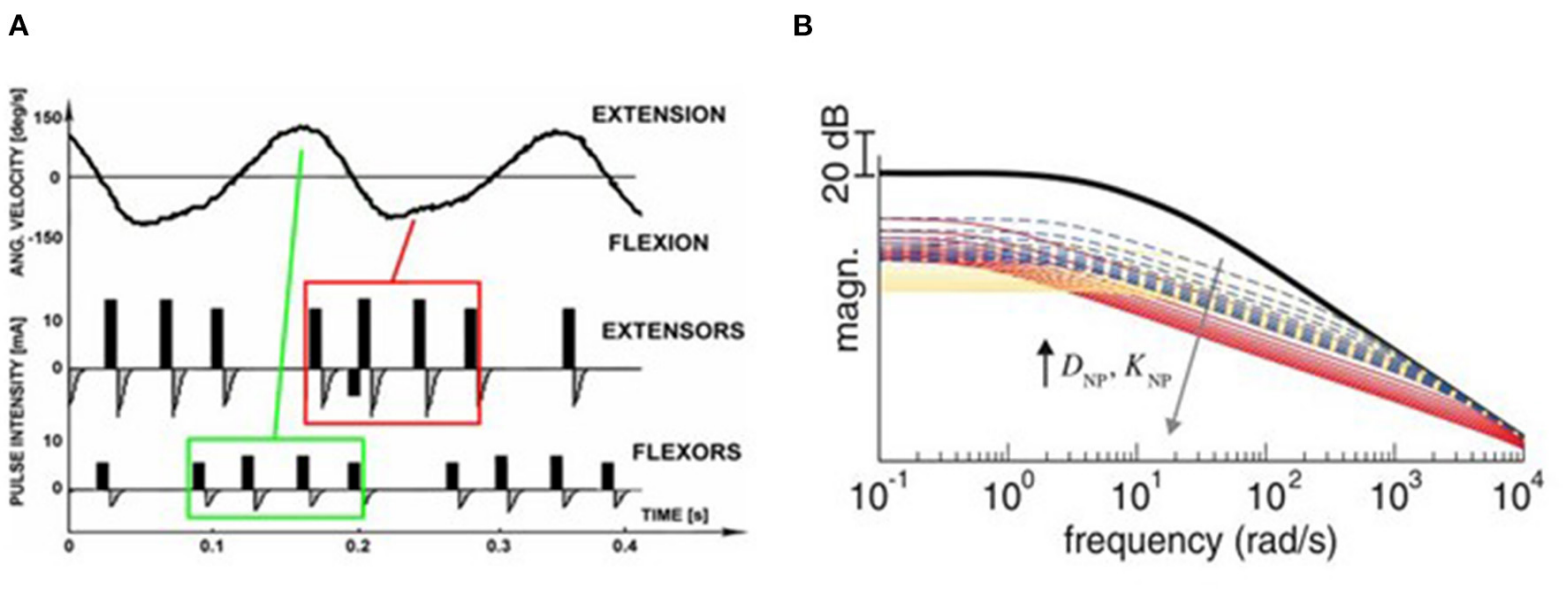
Stimulation strategies for tremor suppression. **(A)** Out-of-phase. The top panel shows the angular velocity of the joint when rest tremor occurred. The antagonist muscle was electrically stimulated when the agonist muscle was active (Maneski et al., 2011). **(B)** Co-contraction. The human joint impendence was modeled with the use of the stiffness and viscosity variables D_NP_ and K_NP_ determined by the stimulation parameters. Equal increments of D_NP_ and K_NP_ are shown as broken lines, larger increments of D_NP_ than K_NP_ are shown as red lines while larger increments of K_NP_ than D_NP_ are shown as yellow lines. The high frequency response of the joint can be attenuated so that the tremor was suppressed (Alvaro Gallego et al., 2013a).

The out-of-phase stimulation activates the antagonist muscle contraction to generate an opposite force for reducing tremor (Maneski et al., 2011), as shown in Figure 2A. Activations of tremorgenic muscles could be characterized using surface EMG signals and utilized as inputs of FES controller to stimulate the pair of antagonistic muscles reciprocally (Zhang and Ang, 2007; Maneski et al., 2011; Zhang et al., 2011). If the neuronal activation of tremorgenic muscles was considered as a disturbance of a close-loop system, it was feasible to suppress the high-frequency tremor-related movement while minimally affecting low-frequency voluntary movement with the use of a proper feedback filter. Dosen et al. (2015) showed that the out-of-phase manner-based system led to a tremor reduction of 46–81% and 35–48%, respectively, while using the stimulation above and below the motor threshold. It suggested that the SES can be an alternative approach for tremor suppression with the advantage of preventing muscle fatigue and sensory discomfort compared to the FES method. Dideriksen et al. (2017) compared surface and intramuscular SES and found that the out-of-phase strategy was effective for both types of stimulation.

The co-contraction stimulation elicited co-contraction of antagonistic muscle pair to increase stiffness and viscosity resulting in significant tremor reduction (Freeman et al., 2015; Copur et al., 2019). The strategy is relatively simple but low joint frequency may affect patients' voluntary movement and the patients might have muscle fatigue quickly as they need to generate more muscle force to perform voluntary activities. Musculoskeletal models have been applied to evaluate the feasibility of FES based on the co-contraction strategy for tremor suppression (Wang et al., 2015; Zhang et al., 2019). The joint can be modeled as a 2nd order linear time variant system in which the FES effect was described as a variable stiffness and viscosity (Alvaro Gallego et al., 2013a,b). As shown in Figure 2B, the frequency response of wrist joint was modified with the increment of co-contraction of the muscle pair.

The continuous stimulation refers to an approach that applies constant current stimulation pulses when tremor detected without following any time blocks or physiological events (Hao M.-Z. et al., 2013; Xu et al., 2016; Hao et al., 2017; Hu Z. et al., 2019; Hu Z.-X. et al., 2019). The approach is easy to be implemented in practice as it does not require a closed-loop feedback control system (Heo et al., 2018). Studies have shown that both FES and SES method with continuous stimulation strategy can significantly attenuate tremor (Jitkritsadakul et al., 2015, 2017; Pascual-Valdunciel et al., 2019). Heo et al. (2018, 2019) discussed the tremor inhibitory effect of sustained sensory stimulation on PD patients with and without scans without evidence of dopaminergic deficit (SWEDD) and found that the strategy can only inhibit PD tremor but not SWEDD-related tremor. However, the stimulation intensity is considered as the most significant parameter as more afferent fibers are expected to be recruited with a higher stimulation amplitude in order to enhance tremor suppression rate (Jitkritsadakul et al., 2015, 2017).

### Electrical Stimulation Locations and Parameters

**Figure 3:**
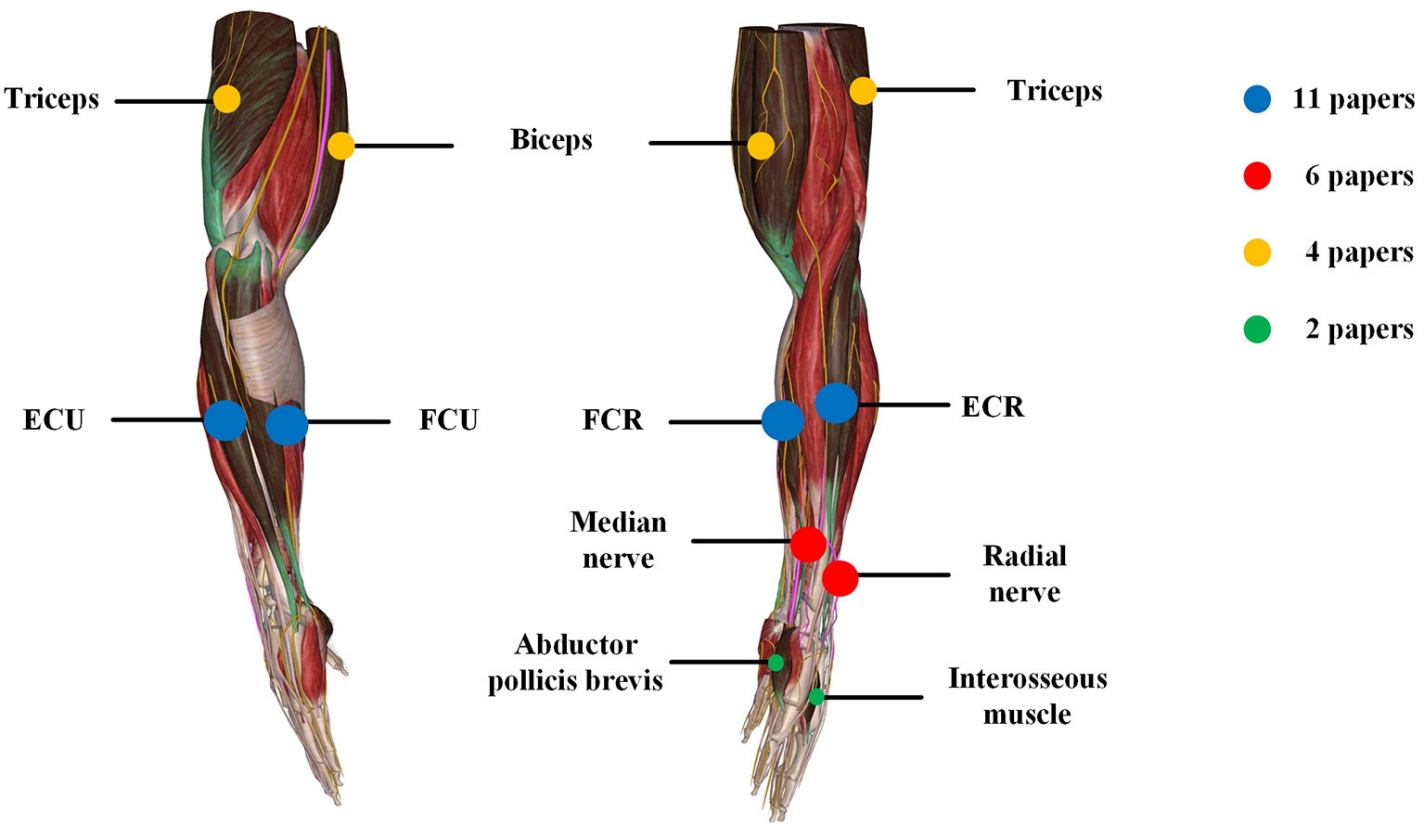
Summary of locations of electrical stimulation from the reviewed articles. The color represents different stimulation location and circle size represents the number of papers.

PD Tremor occurs more often on the upper limb while the wrist tremor is observed most pronounced. The muscles, such as flexor carpi radialis (FCR), extensor carpi radialis (ECR), flexor carpi ulnaris (FCU), and extensor carpi ulnaris (ECU), were usually chosen in the related studies for the FES and SES (Maneski et al., 2011; Zhang et al., 2011; Alvaro Gallego et al., 2013a,b; Dosen et al., 2015; Freeman et al., 2015; Castro et al., 2017; Dideriksen et al., 2017; Heo et al., 2018, 2019; Copur et al., 2019), as shown in Figure 3. However, the tremor may be transmitted to the proximal joints from the distal joints, such as wrist and elbow supination (Davidson and Charles, 2017). The biceps and triceps muscles were electrically stimulated for attenuating the elbow tremor (Zhang and Ang, 2007; Maneski et al., 2011; Zhang et al., 2011; Alvaro Gallego et al., 2013b). Jitkritsadakul et al. (2015, 2017) developed a tremor's glove where electrodes were placed on the abductor pollicis brevis and interosseous muscles. The median and radial nerves were often located for TENS. As the stimulation of the nerves could affect the spinal cord interneurons as the nerves are located beyond the spinal cord (Walsh et al., 1995; Zehr and Kido, 2001), the ECR H-reflex could be observed while the radial nerve was stimulation and the FCR was activated with the stimulation of the median nerve (Pascual-Valdunciel et al., 2019).

**Figure 4:**
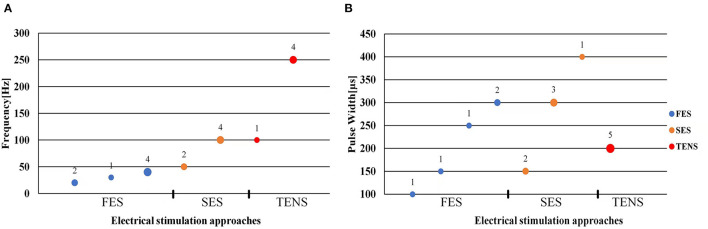
Scatter plot of stimulation frequency **(A)** and pulse width **(B)**. Blue dots represent functional electrical stimulation (FES), yellow dots represent sensory electrical stimulation (SES) and red dots represent transcutaneous electrical nerve stimulation (TENS). The circle size and its superscript indicate the number of studies.

The stimulation frequency and intensity are two essential parameters in the control strategy. The range of stimulation frequency used in the reviewed studies is 20~250Hz (detailed in Tables 1–3). The FES method utilized a relatively lower frequency ranging from 20 Hz (Zhang and Ang, 2007; Zhang et al., 2011) to 40 Hz (Maneski et al., 2011) compared to the SES and TENS as shown in Figure 4A. The minimum and maximum stimulation frequency used for the SES was 50 Hz (Jitkritsadakul et al., 2015) and 100 Hz (Dosen et al., 2015). TENS has a higher frequency range of 100–250 Hz (Hao M.-Z. et al., 2013; Xu et al., 2016).

The stimulation intensity usually varied in participants with a range of 5–30 mA (Zhang and Ang, 2007; Maneski et al., 2011; Dosen et al., 2015; Freeman et al., 2015; Jitkritsadakul et al., 2015, 2017; Xu et al., 2016; Hao et al., 2017; Heo et al., 2018, 2019; Pascual-Valdunciel et al., 2019). For the FES methods, the intensity needs to be set to a level that produces an efficient motor response without causing discomfort (Freeman et al., 2015). The minimum intensity that can elicit muscle contraction is called motor threshold (MT). The stimulation intensity for the SES and TENS is below the MT (Heo et al., 2018, 2019). The amplitude of the maximum M wave was be used as a reference to define the MT (Pascual-Valdunciel et al., 2019). Xu et al. (2016) and Hao et al. (2017) set the stimulation intensity as 1.5 times of radiation threshold measured from the subject for tremor suppression in a close-loop system with the use of SES. A net excitation of motor neurons can be elicited with a higher stimulation frequency and lower stimulation intensity so that muscle fatigue might not be a limitation in this scenario (Dosen et al., 2015).

Bi-phasic current pulse was commonly used (Maneski et al., 2011; Hao M.-Z. et al., 2013; Freeman et al., 2015; Xu et al., 2016; Hao et al., 2017; Copur et al., 2019; Hu Z. et al., 2019; Hu Z.-X. et al., 2019) for avoiding electrochemical imbalance and furtherly tissue damage (Gil-Castillo et al., 2020). The pulse width varies in the studies ranging from 100 to 400 μs, as shown in Figure 4B. The FES methods selected relatively shorter pulse (100–300 μs) while the SES and TENS required a relatively longer pulse (150–400 μs).

### Tremor Detection and Assessment

Various types of sensors, such as motion sensors, mechanical sensors and electrophysiological signals, can be used to detect the hand movement with tremors. Tremor-related features could be distinguished from voluntary movements and identified with the use of machine learning methods. The approaches for tremor detection usually fall into two main categories based on the type of sensor feedback, respectively, electromyography (EMG) -based and inertial-based approach.

**Figure 5:**
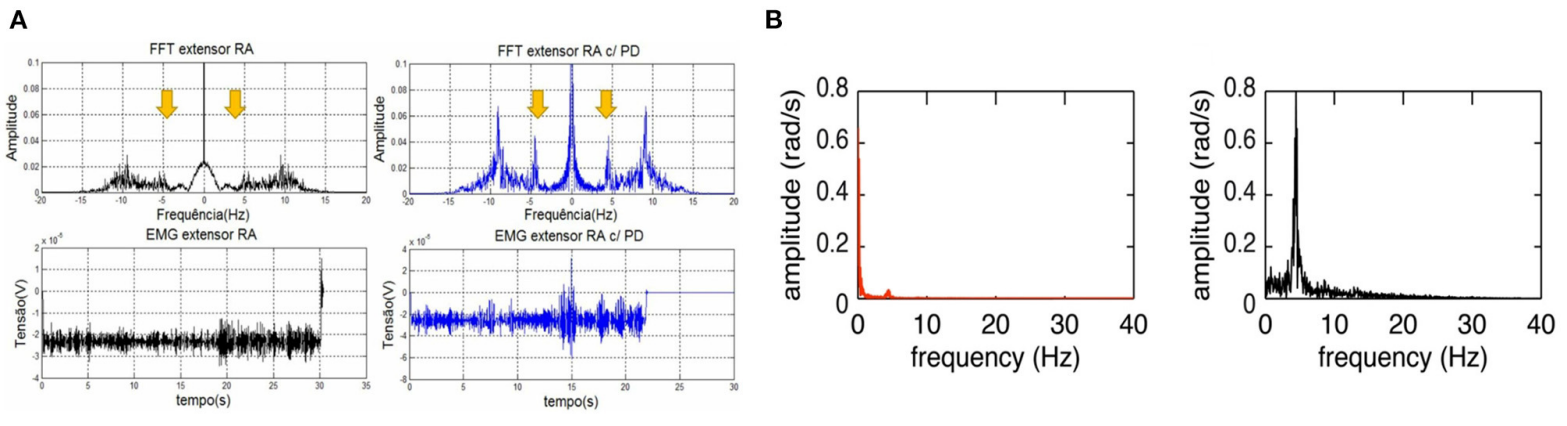
Frequency characteristics of PD tremor based on the EMG signals and inertial data. **(A)** Comparison of temporal and spectral features of EMG signal in the wrist extensor muscle between a PD patient (blue) and healthy person (black). The spectral-domain plot of PD patient's EMG exhibits an additional tremor frequency at 4.55 Hz (Castro et al., 2017). **(B)** Comparison of spectral features of wrist joint angular velocity between resting tremor (black) and voluntary movement (red) in a PD patient. Voluntary movement has a much lower frequency (0–2 Hz) compared to that of Parkinsonian tremor (Lambrecht et al., 2014).

As surface EMG represents the activity of motor neurons reflecting the neural mechanisms in the central nervous system, the tremor EMG signals from one or more antagonistic muscle pairs were investigated while the tremor characteristics (i.e., tremor amplitude, tremor frequency) can be estimated (Dideriksen et al., 2011, 2017). Studies showed that PD tremor exhibits distinguished features in the amplitude- and spectral-domain of EMG signals compared to healthy elders and patients with essential tremor. An additional frequency of 4.55 Hz in PD was observed as shown in Figure 5A (Castro et al., 2017; Karamesinis et al., 2021). The fixed amplitude or frequency threshold based on EMG signal have been commonly used in an online tremor suppression system, however, adaptive threshold can be considered to improve the performance of tremor detection (Dosen et al., 2015; Xu et al., 2016; Hao et al., 2017; Hu Z. et al., 2019). Multilayer perceptron and recurrent neural network models were also used to predict the EMG envelop or raw signals for forecasting the tremor amplitude and frequency used as input in a closed-loop strategy for tremor suppression (Zanini et al., 2019).

Inertial sensors usually consist of an accelerometer and gyroscope measuring the linear acceleration and angular velocity of limb movement which can be used to estimate the tremor amplitude and frequency on the 3-axis dimension (Karamesinis et al., 2021). Motion features, such as root mean square, peak amplitude and frequency of angular velocity or displacement, were used to detect the tremor using set-up thresholds (Jitkritsadakul et al., 2015, 2017; Zhou et al., 2018). In Lambrecht's study (Lambrecht et al., 2014), four IMUs were placed, respectively, on the hand, forearm, upper-arm and humerus segments while angular velocity related features was used to distinguish resting tremor (~5 Hz) from voluntary movements (0–2 Hz), as shown in Figure 5B.

### Clinical Outcomes

Resting tremor is common in PD patients. The patients were usually instructed to place the forearm on a comfortable supporter in a relax posture in the clinical trials (Deuschl et al., 1998). Some studies required patients to perform distracting tasks, such speaking, Eyes closed, counting down, drawing shapes with the contralateral hand, in order to reduce volitionary inhibition of tremor (Feys et al., 2006; Raethjen et al., 2008; Kaski, 2015; Berlot et al., 2021). The time length of experiment trial varies from 15 to 150 s. Five studies recorded data before, during and after electrical stimulation (Hao M.-Z. et al., 2013; Xu et al., 2016; Hao et al., 2017; Heo et al., 2018, 2019). Jitkritsadakul et al. (2015, 2017) compared the tremor parameters with and without stimuli. Maneski et al. (2011) adopted an intermittent stimulation mode named “3 + 1” in which 1 s stimulation pause was taken after 3 s stimulation for updating the estimated tremor frequency and phase. To observe the inhibition of PD tremors during voluntary movement, Hu Z. et al. (2019) and Hu Z.-X. et al. (2019) set a sound hint at 2 s after a 5 s-duration stimulation began when the participants performed a fast-reaching action without visual feedback. Although some studies enrolled different groups (e.g., PD, ET) to perform the same tasks (Maneski et al., 2011; Alvaro Gallego et al., 2013a,b; Dideriksen et al., 2017), Dosen et al. (2015) suggested that the pathophysiology of different tremors should be considered in the experiment set-up.

Clinical outcomes showed high outcome variability in studies as shown in Table 1. The FES methods achieved an average inhibition rate of tremor over 50% (Maneski et al., 2011; Zhang et al., 2011; Alvaro Gallego et al., 2013a,b; Freeman et al., 2015). Zhang et al. (2011) proposed a biological inspired neural oscillator that obtained a maximal tremor suppression rate of 90%. PD and ET patients recruited in Maneski et al. (2011) performed a tremor reduction of 61 ± 7% for all subjects. Dosen et al. (2015) compared SES and FES inhibition rates. The results showed that the tremor suppression with the SES (42 ± 5%) lower than that with the FES (60 ± 14%). However, Dideriksen et al. (2017) demonstrated that SES achieved the average tremor suppression level of 58 ± 35% across all patients. Continuous SES reduced tremors in PD patients by an average of 53–68% but had no effect on SWEDDs patients (Heo et al., 2018, 2019). Study of Hao et al. (2017) showed an average joint angle inhibition rate of 61.56% and an average EMG inhibition rate of 47.97% for all subjects with the use of TENS. A higher optimal tremor suppression rate (60 ± 30%) was obtained in PD patients compared to ET patients (41 ± 34%). Pascual-Valdunciel et al. (2019) compared the outcomes between the stimulation on the ECR and FCR, respectively. The results showed that the ECR inhibition rate is 14 ± 5%−27 ± 9% and the FCR inhibition rate is 57 ± 13%−75 ± 12%, which indicates that SES may be more effective in suppressing the FCR-induced tremor. Overall, the FES method performed a higher rate of tremor reduction compared to the SES and TENS. It needs to be noted that the differences in the assessment methodology, experiment protocol and stimulation strategy might affect the reliability of result comparison among studies.

### Underlying Mechanisms

The three PES approaches have different mechanisms for tremor suppression. However, the mechanisms of tremor reduction using the SES and TENS is still unknown. The application of SES to a muscle may result in the activation of type Ia pathway which excites alpha motor neurons while inhibits the antagonistic muscle (Wargon et al., 2006). Through afferent pathway, the SES might affect the cerebellum (Heo et al., 2018), which was suggested to be one main source of PD tremor (Muthuraman et al., 2012, 2018). The TENS modulates spinal stretch reflex via type Ib inhibition (Pierrot-Deseilligny, 1996; Hao M. et al., 2013; Hao M.-Z. et al., 2013). The peripherical proprioceptive sensory fibers were stimulated while the excitation modulates the transmission of sensory signals from the peripherical nervous system to the central nervous system (Hao M. et al., 2013). Proprioceptive sensory feedback is carried into thalamic circuit that is hypothesized to be involved in PD tremor generation. Therefore, the SES and TENS showed a higher tremor suppression rate in PD patients compared to patients with other pathological tremors, such as ET and SWEDD (Dosen et al., 2015; Heo et al., 2019). The mechanisms for mechanical tremor suppression based on the FES are well-understood. With trains of low-level electrical current pulses activated extensor and flexor muscles, muscle torques that are opposite to the tremor-related muscle torques will eliminate PD tremor (Javidan et al., 1992; Heroux et al., 2010). The FES can obtained an optimal tremor suppression rate (maximum rate > 90%) with an advanced close-loop controller (Zhang et al., 2011).

### Limitations and Perspectives

These studies demonstrated the feasibility and great potential of PES as an efficient intervention for tremor suppression in PD patients. However, we also see several limitations in current research: Firstly, the stimulation methods and parameters were very different in the studies leading to high variability in clinical outcome results. Most studies utilized a feedback or feedforward controller in which the input was usually EMG signal or inertial data and the onset was switched on/off with a tremor detector. The controller performance determines the outcome of tremor suppression. Secondly, the sample size is relatively small while lacking control and pseudo-groups. Therefore, a standardized and cross-section study with large groups is significant for quantifying the effect of tremor attenuation on PD patients.

## Conclusion

This paper systematically reviewed 20 original studies for PD tremor suppression based on PES methods in recent 10 years. The results show that the PES is an efficient approach for tremor suppression. The methods fall into three main categories according to the mechanisms: namely FES, SES and TENS. The effect on tremor suppression can be varied due to stimulation approach, electrode location and stimulation parameters. The FES method performed the best in tremor attenuation; however, the tremor suppression efficiency is determined mainly by the control strategy and accuracy of tremor detection. The mechanism underlying tremor suppression with sensory and peripherical nerve stimulation is not well-known. Current stimulation approaches could only work for a part of patients. The potential mechanism of tremor suppression still needs to be further explored.

## Data Availability Statement

The original contributions presented in the study are included in the article/supplementary material, further inquiries can be directed to the corresponding authors.

## Author Contributions

LM, XZ, and DM: conception and design of the study. MJ: acquisition of literature data. LM and MJ: analysis and interpretation of data and drafting the manuscript. XZ and DM: revising the article critically for important intellectual content. All authors approved the final version to be submitted.

## Funding

This study was supported by the National Natural Science Foundation of China (82001921), the National Key R&D Program of China (2020YFC2004300, 2020YFC2004302) and the Natural Science Foundation of Tianjin (20JCZDC0080).

## Conflict of Interest

The authors declare that the research was conducted in the absence of any commercial or financial relationships that could be construed as a potential conflict of interest.

## Publisher's Note

All claims expressed in this article are solely those of the authors and do not necessarily represent those of their affiliated organizations, or those of the publisher, the editors and the reviewers. Any product that may be evaluated in this article, or claim that may be made by its manufacturer, is not guaranteed or endorsed by the publisher.
